# A new perspective on the proper timing of radiotherapy during CDK4/6 inhibitor therapy in patients with “bone-only” metastatic breast cancer

**DOI:** 10.3389/pore.2023.1611369

**Published:** 2023-10-11

**Authors:** Ilona Tornyi, Peter Árkosy, Ildikó Horváth, Andrea Furka

**Affiliations:** ^1^ Department of Pulmonology, Faculty of Medicine, University of Debrecen, Debrecen, Hungary; ^2^ Department of Oncology, Faculty of Medicine, University of Debrecen, Debrecen, Hungary; ^3^ Department of Clinical Radiology, Faculty of Health Care, Institute of Practical Methodology and Diagnostics, University of Miskolc, Miskolc, Hungary

**Keywords:** breast cancer, radiotherapy, radiosensitivity, CDK4/6 inhibitors, treatment timing

## Abstract

The vast majority of hormone positive and HER2 negative advanced breast cancers can be controlled well by endocrine therapy combined with the groundbreaking use of CDK4/6 inhibitors in the metastatic first-line setting. Approximately 50%–60% of these patients have “bone-only” metastatic disease. In oligometastatic cases or if a certain number of uncontrolled lesions develop during the aforementioned therapy, ablative radiotherapy can be delivered or, in symptomatic cases, urgent irradiation is needed with palliative intent. To achieve the most effective results, parallel with good quality of life, the timing of radiotherapy must be determined precisely, taking into account that different cell cycles are involved during different treatment modalities; therefore, optimization of treatment schedules ensures longer and safer post-progression overall survival. The key question is whether the two treatment modalities are safe concurrently or whether they should be administered separately, and if so, what is the optimal sequence and why? This manuscript aims to answer this important question, with a focus on quality of life. Existing publications focus on safety and toxicity profiles, and efficacy is detailed only tangentially and minimally.

## Introduction

Breast cancer is the most frequently diagnosed malignancy and the leading cause of cancer-related mortality in women, worldwide [1]. The vast majority, approximately 75% of breast cancer patients, belongs to hormone-positive subtypes, with a relatively good prognosis [2]; thus, endocrine therapy is a highly effective treatment for these patients. Approximately 30% of women with an early-stage breast cancer diagnosis develop metastasis [3]. The metastatic site depends on the pathological subtype of the primary tumor, but in breast cancer, 30%–60% have metastases in the bone, 21%–32% in the lung, 15%–32% in the liver, and 4%–10% in the brain based on the SEER database [4]. Only 13% of primary breast cancer patients who developed metastasis in the bone survived >5 years in a Danish population-based cohort study [5].

New targeted drugs had to be developed to treat such cases. In cases of hormone receptor (HR)-positive and human epidermal growth factor receptor 2 (HER2)-negative breast cancer, based on risk factors, selective cyclin-dependent kinase 4/6 (CDK4/6) inhibitors are widely used to postpone the need for chemotherapy. Up until now, three CDK4/6 inhibitors have been approved for the treatment of HR-positive advanced or metastatic breast cancer [6]. Palbociclib was the first authorized by the European Medicine Agency in November 2016 based on PALOMA studies [7], followed by ribociclib in August 2017 based on MONALEESA studies [8] and abemaciclib in September 2018 based on MONARCH studies [9]. These drugs are generally used in combination with hormone replacement therapies, considering current menopausal status, although abemaciclib was also approved as monotherapy. The latter was also approved for node-positive, high recurrence risk, Ki67 score ≥20% HR+ luminal B type breast cancer patients in adjuvant settings in October 2021 based on a monarchE study [10]. Beyond these, there are further drugs on the horizon. Dalpiciclib, which has good penetration of the blood-brain barrier, based on a DAWNA-1 randomized phase 3 trial [11], was conducted only in Chinese subjects, so its performance in other populations is still an open question. GLR2007 is also a promising new CDK4/6 inhibitor [12] that is effective in smaller concentrations compared with abemaciclib, which also has proper blood-brain barrier permeation. This suggests fewer adverse events and more effective treatment in cases of central nervous system metastasis. Lerociclib accumulates better in xenografts in preclinical studies; thus, fewer hematological and gastrointestinal side effects are presumed to be observed. Trilaciclib is also a promising new drug, which differs from all the above-mentioned drugs due to its intravenous administration route [13].

Approximately 50%–60% of hormone-positive and HER2-negative advanced breast cancer patients have “bone-only” disease [14]. Depending on the number, site, and size of metastasis, radiotherapy is often suggested as a local treatment modality to dominate symptoms or block tumor growth. Radiation can be delivered with palliative intent, e.g., pain relief, or even with local ablative intent to destroy certain small-size metastasis, mainly in oligometastatic settings. The main question is whether the two treatment modalities are safe concurrently or should be administered separately, and if so, what is the optimal sequence and why? This study investigates this pressing question from a molecular biology point of view and how we can implement the results, based on preclinical studies [15–17], into daily clinical practice.

## Molecular biology aspects of certain breast cancer treatments

### Radiotherapy

More than 100 years passed between the first breast radical mastectomy being performed and the development of targeted therapies in breast cancer treatment [18]. As we understand more about the biology of cancer, more sophisticated treatment methodologies will become available. Since there are still gaps in our knowledge, we are only able to achieve partial success with our commonly available (surgery, radiotherapy, chemotherapy) and more frequently used (hormone, immunotherapy, targeted therapy) tools.

Radiotherapy (RT) is widely used in cancer treatment. The first utilization of RT was in the early 20th century. RT treatment modalities—from superficial X-ray to ultra-high dose rate FLASH radiotherapy—have changed substantially [19], yet the main radiobiology concept remains the same. Ionizing radiation has direct and indirect effects by producing water radicals on DNA chains. Both causes mainly double-strand break and single-strand lesions, respectively [20, 21]. When DNA suffers irreparable damage, a damage signal cascade starts involving ATM, Chk2, and p53 proteins and stops cell cycles at the G1/S phase [22]. When DNA suffers single-strand break DNA damage, another pathway starts involving ATR, and Chk1, and this stops the cell cycle at the G2/M checkpoint [23]. Cancer cells often lose normal p53 function and cannot stop at the G1/S checkpoint. In this case, cells can only use G2/M checkpoints, while normal cells can stop and repair DNA at the G1/S phase.

Cells have a different sensitivity to ionization exposure that leads to different radiation-induced cell death [24]. The most sensitive phase of radiotherapy is mitosis.

Radiotherapy is not target selective, it acts on rapidly growing cancer and normal cells, too, but we can modulate its fraction, energy, and direction. Novel technological advances, such as intensity-modulated and image-guided radiotherapy were improved to deliver therapeutic doses and boost the gross tumor volume (GTV), thus increasing the difference between tumor control probability and normal tissue complications. Other advances, such as stereotactic body radiotherapy (SBRT), have enhanced dose conformity to substantially limit treatment margins, so a critical ablative dose of irradiation can be delivered to the tumor in one (radiosurgery) or in several fractions [25].

### CDK4/6 inhibitors

Cell cycle is a highly ordered system, driven by cyclin-dependent kinases (CDKs). Cyclin-dependent kinase inhibitors target overactive CDKs. CDK4 and CDK6 have two key functions. In a complex with cyclin-D, CDKs phosphorylate retinoblastoma (Rb) protein, a tumor suppressor protein that activates E2F transcription factor, and sequestering cell cycle inhibitors p21Cip1 and p27Kip1 proteins, which leads to activation of cyclin-E dependent CDK2 containing complexes [26]. Cyclin-D has three isoforms: cyclin-D1, cyclin-D2, and cyclin-D3. Isoforms have different lengths; cyclin-D1 is 295 (UniProt:P24385), cyclin-D2 is 289 (UniProt:P30281), and cyclin-D3 is 292 (UniProt:P30279) amino acids long. The phosphorylation site for ubiquitination is also different in cyclin-D1 at Thr-286, in cyclin-D2 at Thr-280, and in cyclin-D3 at Thr-283 position. These isoforms form a heterodimer complex with CDK4/6 and in this complex can phosphorylate and, thus, inactivate Rb.

CDK4/6 inhibitors bind to the ATP domain of CDK4/6 but have different cyclin-D targets. Palbociclib inhibits D1-CDK4, D2-CDK6, and D3-CDK4. Ribociclib attacks D1-CDK4 and D3-CDK6. Abemaciclib targets D1-CDK4 and D1-CDK6 [17].

Mechanistically, pharmacological CDK4/6 inhibition prevents the phosphorylation of downstream cell cycle proteins, such as Rb, that control cell cycle progression through the G1/S checkpoint [27]. Inhibition of CDK4 and CDK6 induces cell cycle arrest at the G1/S checkpoint.

In breast cancer, active cyclin-D in a complex with CDK4/6 is considered to play a high-impact role in estrogen-driven cell proliferation [28, 29]. In clinical practice, novel FDA-approved CDK4/6 inhibitors are used in breast cancer treatment [30].

**FIGURE 1:**
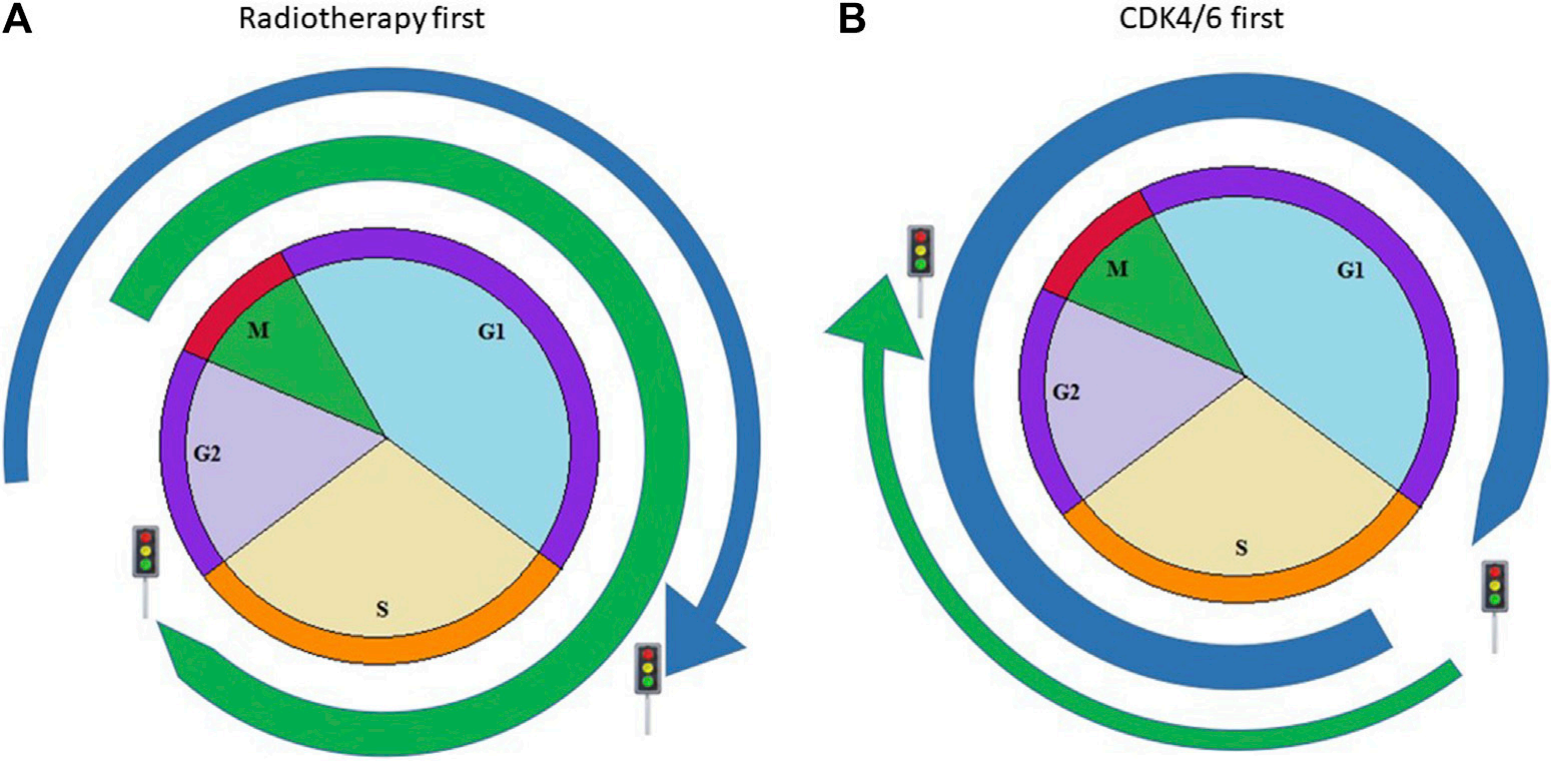
Possible combination of CDK4/6 inhibitors with radiotherapy. Blue arrow simulates the effect of CDK4/6 inhibitors according to cell cycles, green arrow shows the acting point of radiotherapy in cell cycle. **(A)** Figure shows the case when radiotherapy is delivered before CKD4/6 inhibitors and acts in the M phase, resulting in more cancer cell deaths **(B)** Figure shows the case when CDK4/6 inhibitors block cancerous cells in an earlier phase; therefore, tumorous cells cannot reach the M phase, which is the most sensitive phase to radiotherapy.

A combination of common and new treatments is recommended in clinical guidelines. However, the ideal treatment combination and timing to reach their synergistic effect are not yet clear. CDK4/6 inhibitors and other DNA-damaging therapies, like certain chemotherapies or radiotherapy, need proper therapeutic order as they target different cell cycle phases. If CDK4/6 inhibitors arrest the cycle in the G1/S or G0/G1 phase, it prevents the cells from entering the subsequent phase. An antagonistic effect occurs as cell cycles do not enter into their next phases, so radiotherapy cannot develop its complete effect [13]. If irradiation is required and CDK4/6 inhibitors should be suspended, the elimination time of different drugs must be taken into account (Figure 1).

Pharmacokinetics of CDK4/6 inhibitors are slightly different. Their metabolism is through CYP3A4 enzyme, and palbociclib also interacts with SULT2A1. Their half-life times are also different: 24–34 h of palbociclib, 30–55 h of ribociclib, and 17–38 h of abemaciclib. Within 3 days, they half, suggesting a 1 week drug-free period before irradiation [12].

### CDK4/6 and radiotherapy *in vitro*/*in vivo* studies

Pesch et al. tested the effect of CDKi alone and in combination with RT on ER+ breast cancer cell lines and in mouse xenografts. They conclude that in radiosensitized cell lines, CDKi suppresses cell cycle signaling and changes the DNA repair response. In mouse xenograft models, concurrent administration of CDKi and RT suppressed the tumor growth and prolonged tumor doubling time compared to controls that received monotherapies [31]. In a recent study, Klapp et al. tested a sequenced RT and CDKi treatment and measured its optimal schedule through cellular senescence. They demonstrated that the RT-first approach induced an increased level of cellular senescence in cell lines and a mouse model, respectively [32].

The bone-only metastatic breast cancer treatment cannot be modeled in preclinical cell culture or in animal experiments as it is special circumstance.

### CDK4/6 and radiotherapy clinical trials

The main difference between the two modalities is that radiotherapy stimulates cells toward certain cell death, while CDK4/6 inhibitors block cell cycle progression at the G1/S checkpoint. Pretreated cells by CDK4/6 inhibitors theoretically are less sensitive to radiotherapy as they are blocked at G1/S.

Few trials have investigated the effects of CDK4/6 inhibitors alone or in combination with radiotherapy according to ClinicalTrials.gov. There are only five ongoing or recruiting clinical trials investigating CDK4/6 inhibitors with radiation therapy in breast cancer with bone metastases (Table 1).

**TABLE 1:** Ongoing clinical trials of CDK4/6 inhibitors in combination with radiotherapy in metastatic breast cancer.

Trial number/name	Phase	Enrollment criteria	Fractionation	Drug	Association RT/CDK4/6inhibitors	Treatment sequence	Recruitment status	Planned enrollment	Actual enrollment
NCT03691493	Phase 2	*de novo* bone metastasis	Conventionally	Palbociclib	Concurrent	-	Active, not recruiting	42	38
ASPIRE									
NCT05334459	Phase 1	*de novo* oligometastatic	Conventionally	CDK 4/6i	Sequential	N.A.	Recruiting	40	N.A.
ISTMET									
NCT03870919	N.A.	*de novo* bone-only	Conventionally	Palbociclib	Sequential	RT after 6 cycles systemic treatment	Recruiting	200	N.A.
PALATINE									
NCT03750396	Phase 2	*de novo* metastatic	Mainly SBRT	CDK 4/6i or mTORi	N.A.	N.A.	Recruiting	110	N.A.
CLEAR									
ACTRN12620001212943	Phase 2	*de novo* oligometastatic	SBRT	CDK4/6i	Sequential	Radiotherapy in CDK4/6 pause	Recruiting	32	N.A.
AVATAR									

In an NCT03691493 phase 2 trial, CDK4/6 inhibitor (palbociclib) in combination with a nonsteroidal aromatase inhibitor and radiation therapy was used; in an NCT03870919 trial, only a locoregional treatment with palbociclib was used; and in NCT04923542 phase 1 & 2 trials, abemaciclib in combination with endocrine therapy and stereotactic radiosurgery was used. According to the currently recruiting AVATAR phase 2 study protocol, CDK4/6 inhibitors must be stopped at least 3 days prior to SRT and allowed to continue 3 days following STR completion. The authors also declare that a week off after CDK4/6 inhibitor treatment is optimal if feasible [33].

Besides breast cancer, there are trials investigating the effects of CDK4/6 inhibitors in prostate cancer, head and neck cancer, and gliomas.

### Toxicity of radiotherapy and CDK 4/6 inhibitor therapies

Meattini et al. reported preliminary experience with five metastatic breast cancer patients who received concomitant ribliciclib and palliative RT. Two of five patients had G3-4 adverse events (neutropenia, vomiting, and diarrhea) [34]. David et al. reported five cases with G2-5 adverse events (pneumonitis, dermatitis, oesophagitis) during palbociclib and RT concurrent or separate treatment [35]. A safety and feasibility study conducted on 288 ER+ advanced breast cancer patients used RT and CDKi sequentially and concurrently. This retrospective study mainly focused on severe adverse events. They found that neutropenia was more frequent in a concurrent treatment arm [36]. Based on these studies, concurrent treatment has the potential to result in more adverse events.

The concurrent use of CDK4/6 inhibitors and radiotherapy may indeed lead to an increased risk of side effects. Both CDK4/6 inhibitors and radiotherapy can individually cause side effects, and their combination can potentially amplify these. However, it is important to note that the specific side effects and their severity can vary depending on the individual patient, specific drugs, and radiation protocols used. When CDK4/6 inhibitors and radiotherapy are used concurrently, there can be overlapping or additive effects on the body, potentially leading to increased side effects.

## Translational implications/clinical applications

The optimal timing of radiotherapy and CDK4/6 inhibitor plus endocrine partner therapy administration is translational implication; therefore, it could be a major benefit from the view of clinical application [16].

In drug registration clinical trials [37–40], irradiation was allowed during CDK4/6 inhibitor therapy. In a PALOMA trial, only 1.9% of the randomized subjects received radiotherapy [7, 41, 42] and it was highlighted that in cases of central nervous system metastasis treated with irradiation, CDK4/6 inhibitors were prohibited.

The more metastatic treatment lines we indicate, the shorter the progression-free survival time. Therefore, it is very important to maintain effective treatment albeit it could not dominate a certain number (less than three) lesions. Thus, with the implementation of impressive local ablative treatment, there is no need for drug switching. This is a paradigm shift in oncology: we understand that malignancies have heterologous clone populations and just those can skip from treatment control whose have acquired resistance. Therefore, except for these lines, properly chosen therapy can block sensitive clones and can be continued safely and undisturbed. Uncontrolled lines can be treated by local ablative treatment methods. If the required result appears on re-staging, i.e., the blockade or destruction of the so-far uncontrolled tumor lesions, the post-progression overall survival time can be prolonged. Significant benefits and overall survival improvement are expected. In such a case, there is no need to modify further therapeutic lines; therefore, good quality of life can be maintained by postponing new drug introduction. Patients can gain time, with a considerably improved and extended lifespan.

Moreover, the latest studies have revealed that CDK4/6 inhibitors may stimulate the immune system, namely, CD8 T-cell immune memory cells, interact with the tumor microenvironment, and have antitumor immunity effects [15, 43].

Radiotherapy is also an immunogenic treatment modality as it causes stress-driven regulated cell death; nowadays, so-called immunogenic cell death is an adaptive immune response [15, 21, 44]. It sensitizes the microenvironment before immune therapies [45], so the primary given radiotherapy may not just be synergistic but may even be a supra-additive effective treatment, taking into account that CDK4/6 inhibitors also affect the immune response.

## Discussion

The toxicity profile of concomitant CDK4/6 inhibitor therapy with irradiation is still unclear, though there are few preclinical studies [30, 45, 46] targeting this question. There are a few that conclude the radiosensitization effects of CDK4/6 inhibitors, although not in every tumor type, mainly in HPV-positive, high-mitotic activity, and head and neck squamous cell cancer, whose biological features are different from relatively slow-growing hormone receptor-positive breast cancers. These studies speculate that the CDK4/6 inhibitor probably blocks cell cycle progression into the radioresistant S phase, but these conclusions have not been substantiated in daily clinical practice [47].

Following exhaustive publication searches, few case series and reports were found to be available, involving few subjects and without control groups, focusing mainly on toxicity profiles with controversial results. Furthermore, the efficacy results of concomitant CDK4/6 inhibitor therapy with irradiation are not detailed. A recently published clinical investigation within a multi-institutional safety and toxicity study reported higher toxicity rates when these two modalities were administered concurrently. According to this clinical investigation, safety and toxicity results appear to be clear. Therefore, the question remains as to which administration schedule is more favorable.

The sequence and timing of different therapies are crucial to reach optimal antitumor effects [16]. The main difference between radiotherapy and CDK4/6 inhibitors is their cancer control: radiotherapy drives cells to certain cell death, while CDK4/6 inhibitors block the cell cycle at the G1/S checkpoint. Pre-treated cells by CDK4/6 inhibitors are theoretically less sensitive to radiotherapy as they block at G1/S. In case of proper order palliative effect is more favorable; therefore, a longer progression-free survival and a better quality of life can be achieved.

The authors suggest the following order in those cases when the delivery of radiotherapy and CDK4/6 inhibitor treatment are both indicated.

If *de novo* diagnosed metastatic breast cancer requires CDK4/6 inhibitor therapy, start with irradiation and then administer CDK4/6 inhibitors and endocrine replacement therapy [48].

When a patient is under treatment with CDK4/6 inhibitor, there are two options depending on the aim and intent of high-energy radiotherapy. In the first case, if palliative ultra-hypofractionated radiation is planned due to severe pain relief, or if there is a risk of fracture or compression caused by bone metastasis that cannot be treated with local ablative dose, 8Gy in a single fraction can be scheduled. Radiotherapy can be given at the end of the drug therapeutic gap (21/7 days schema) in the case of palbociclib and ribociclib. Nota bene, the abemaciclib schedule is continuous; therefore, it can be suspended and radiotherapy can be implemented a week later.

In the second case, in oligometastatic disease or in certain non-responder foci or few (less than three) newer lesions, where treatment with a curative dose is planned, even with stereotactic body radiation or intensity-modulated radiotherapy (IMRT) or volumetric modulated arc therapy (VMAT) with simultaneous integrated boost (SIB), the radiation can be initiated a week after the last pastille intake of CDK4/6 inhibitor. Following the suspension of CDK4/6 inhibitors and completion of radiotherapy, it can be re-administered safely and allowed to continue if restaging radio-biological images show no progression. The re-evaluation of radiological images has to be conducted using the same method used for diagnosis and must be scheduled 6–8 weeks following the completion of radiotherapy. In the case of metabolic information that can be measured on PET/CT, the maximum standardized uptake value (SUVmax) can show the activity changes (hopefully decay) of the tumor.

## Conclusion

After publications and clinical trials were investigated, from molecular biology and clinical aspects, all these findings suggest a new treatment perspective is necessary.

We can conclude that CDK4/6 inhibitor therapy combined with radiotherapy is safe and the authors recommend this therapy for better and more effective outcomes appropriate sequence, which is not written in the official summary of product characteristics. Radiotherapy should be the initial treatment, followed by CDK4/6 inhibitors in *de novo* discovered cases; otherwise, a short suspension of the drug is advised to eliminate the CDK4/6 inhibitor from plasma and let the radiotherapy destroy cancerous cells arriving in the G2/M phase.

The adequate local ablative treatment in oligo-progression could be sufficient and, following this, we can maintain the otherwise effective systemic therapy. Therefore, we can extend and prolong the time interval of the metastatic first-line treatment with CDK4/6 inhibitor, even changing the endocrine partner (letrozole to fulvestrant). This is why it is important to ensure an effective local ablative treatment with radiotherapy in bone-only oligo-metastatic cases. If necessary, bone modifying agent can be shifted (bisphosphonate to denosumab) in another step. In the case of infectivity of these treatment changes, the main component of the first-line treatment (CDK4/6 inhibitor) should be relied on in the second-line treatment. With this soft change, patients can gain time and a good quality of life as we know that further line treatments are usually associated with more adverse events and lead to shorter progression-free intervals. In our daily practice, we deliver SBRT (stereotactic body radiation therapy) for two main reasons: stereotactic radiotherapy doses are potentially more lethal to metastases and may dominate the disease; meanwhile, SBRT requires a shorter treatment time.

Further questions can be addressed by scientists, such as “What about particle radiotherapy combined with targeted agents?” Preliminary data suggest that high LET radiation, such as heavy ion radiation like C ion irradiation may be a favorable partner to CDK4/6 inhibitor application [49]. Probably, this is the future hope when high-energy heavy ion radiation would be the essential part of cancer treatment and wide spread would be available [50].

All oncological treatment must be discussed in a multidisciplinary team and individualized options must be calculated, taking into account further possibilities and as far as possible, maintaining an effective, highly tolerable therapy for as long as we can with the hope of prolonging the good quality of post-limited-progression life.
